# The role of artificial intelligence in the authorship of scientific articles in dentistry: ethical approaches for responsible use

**DOI:** 10.21142/2523-2754-1401-2026-269

**Published:** 2025-12-28

**Authors:** Luis Ernesto Arriola-Guillén

**Affiliations:** 1 Division of Orthodontics, School of Dentistry, Universidad Científica del Sur, Lima, Perú. luchoarriola@gmail.com Universidad Científica del Sur Division of Orthodontics School of Dentistry Universidad Científica del Sur Lima Peru luchoarriola@gmail.com

A central challenge in contemporary dentistry is determining how to ethically and responsibly integrate artificial intelligence tools, such as ChatGPT, DeepSeek, and Google Gemini, into scientific article writing, review, and development, while ensuring that these technologies do not compromise researchers’ originality or fundamental responsibilities ^1^^-^^4^. The coming years will be critical for establishing clear boundaries. Although AI tools can support specific aspects of research, they should not be used to generate research ideas or to author scientific articles. These issues require us to clarify the distinct roles of researchers and establish effective strategies to safeguard the originality of our studies ^5^.

Technology cannot replace human expertise. It lacks the common sense, clinical judgment, and emotional intelligence needed to interpret the diverse research topics in dentistry and its specialties.

Rather than rejecting or fearing technological advancements, researchers should acknowledge their essential role in contemporary research and employ them responsibly to refine scientific communication. These tools can improve writing quality and enhance reader comprehension, particularly given the predominance of English in scientific discourse. Nevertheless, we must avoid becoming conduits for technological plagiarism.

Likewise, specific applications are designed to replace aspects of peer review, yet human judgment remains indispensable due to the unique clinical insights required in each specialty. While these tools can assist by checking for similarity and providing checklists to support reviewers, they serve to enhance the peer review process rather than replace the critical role of the reviewer.

In conclusion, dental researchers should embrace technology and apply it ethically, consistently prioritizing clinical judgment and practical experience as the most valuable components of the research process.
